# An Elderly COVID-19 Patient with Community-Acquired *Legionella* and *Mycoplasma* Coinfections: A Rare Case Report

**DOI:** 10.3390/healthcare9111598

**Published:** 2021-11-21

**Authors:** Sari T. S. Alhuofie

**Affiliations:** Medical Laboratories Technology Department, College of Applied Medical Sciences, Taibah University, Al-Madinah Al-Munwarah 42353, Saudi Arabia; shoufie@taibahu.edu.sa

**Keywords:** SARS-CoV-2, coinfection, community-acquired pneumonia, *Legionella pneumophila*, *Mycoplasma* *pneumoniae*, atypical bacteria

## Abstract

The combination of severe acute respiratory syndrome corona virus-2 (SARS-CoV-2) infection and other respiratory pathogens is a real challenge for health care systems in terms of diagnosis, treatment, and management. Most of the reported bacterial coinfections among SARS-CoV-2 patients are hospital-acquired infections that occurred after several days of hospitalization. Little is known about the incidence of community-acquired atypical bacterial coinfections with SARS-CoV-2. In this work, we report on a rare case of an elderly SARS-CoV-2 patient with underdiagnosed bacterial coinfections who received care in the medical ward for 23 days then was discharged home. Retrospective serological investigation revealed positivity for *Legionella pneumophila* and *Mycoplasma pneumoniae*, indicating double community-acquired atypical bacterial coinfections that were in agreement with clinical manifestations that patients showed at his admission to the hospital. Screening for possible community-acquired respiratory co-pathogens among elderly SARS-CoV-2 patients is critical for effective treatment and management.

## 1. Introduction

Severe acute respiratory syndrome coronavirus 2 (SARS-CoV-2) is a highly contagious respiratory disease that spreads from human to human. It was first detected in Wuhan, China, in December of 2019, and then it spread globally to become a pandemic in less than three months [1]. In Saudi Arabia, the first reported case of COVID-19 infection was on 2 March 2021; since then, cases have exceeded 544,449 patients, including 532,850 recovered cases and 8545 deaths as of 1 September 2020 [2].

Community-acquired pneumonia (CAP) is an infection acquired outside hospitals and might require hospital admission and intensive care. Increasing incidences of CAP occur among the elderly, as they are more susceptible to this infection, which is attributed to several factors such as incompetent immunity, decreased mucociliary function, cardiopulmonary dysfunction, and comorbidities [3]. Early surveillance by May and colleagues found CAP as the third most common hospital diagnosis for patients aged 65 years or older [4]. Several microorganisms, including bacteria and viruses, can cause this infection. The most common causatives are *Streptococcus pneumoniae*, influenza A, *Mycoplasma pneumoniae*, and *Chlamydophila pneumoniae*; other organisms such as *Legionella pneumophila*, *Haemophilus influenza*, and *Staphylococcus aureus* are less common [5].

Coinfecting pathogens with SARS-CoV-2, such as bacteria, viruses, and fungi, have been reported by several observations, and the combination of COVID-19 and other respiratory pathogens can increase the difficulty of diagnosis and treatment and may even accelerate disease symptoms and mortality [6,7,8,9]. Atypical bacteria, including *Mycoplasma* spp., *Legionella*, and *Chlamydia* spp., have been rarely detected during the current COVID-19 pandemic despite their occurrence during the SARS-CoV-1 and MERS-COV endemics [10,11,12]. Only three cases were reported among 5700 COVID-19 patients in the U.S., and zero cases were reported in a cohort study among 162 SARS-CoV-2 patients in Germany [13,14]. However, Xing and colleagues had reported few cases of *M. pneumoniae and L. pneumophila* coinfections among critically ill COVID-19 patients in China [15]. Little is known about atypical bacterial coinfection incidence among COVID-19 patients in Saudi Arabia.

In this case report, a SARS-CoV-2 elderly patient with double atypical bacterial coinfections (*Legionella pneumophila* and *Mycoplasma pneumoniae*) is reported to emphasize the need to consider these infections, especially in elderly SARS-CoV-2 patients.

## 2. Case Description

A 76-year-old man arrived at Al-Madinah General Hospital, King Salman Medical City in Saudi Arabia, on 21 June 2020. He was diagnosed with a COVID-19 infection in the primary health care center three days before his arrival at the hospital as nasopharyngeal swabs were taken from him. Three of his family members were then subjected to a quantitative reverse-transcription polymerase chain reaction (RT-qPCR), revealing positive results.

**Figure 1:**
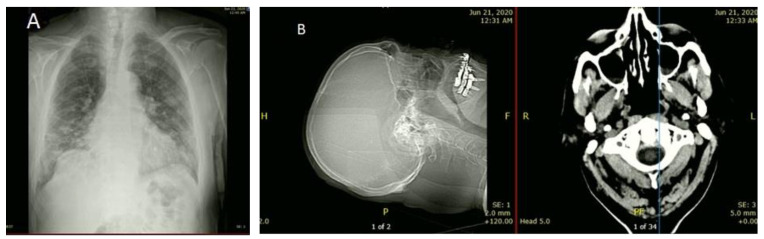
(**A**) Chest X-ray depicting bilateral multifocal patchy opacity more at lower zone and no cardiomegaly. (**B**) Brain CT scans showed no acute major infarction or hemorrhage.

He reported having shortness of breath for 6 days; a fever for a month, which had worsened in the last 2 days (39.4 °C); blood pressure was 133/75 mmHg, suggesting no hypertension; heart rate was 78 beats/min; and respiratory rate was 22 cycles/min. A productive cough with yellow sputum, loss of appetite, and a change in his sense of taste was recorded. He reported no diarrhea, sore throat, or previous chronic disease. He was conscious, oriented to time, place, and person, and his SiO_2_, which was 77% at the time of his arrival, corrected to 94% on 4 L O_2_. His chest X-ray revealed bilateral multifocal patchy opacity, more in the lower zone, and no cardiomegaly. Brain CT scan showed no acute major infraction or hemorrhage (Figure 1), and the ECG displayed no significant changes. Laboratory investigations results at admission showed an Hb1AC level of 7.10, indicating diabetes and increased prothrombin time of 14 s. In addition, WBC was 12,030/μL, revealing neutrophilia (11,340/μL) and lymphopenia (370/μL) (Table 1). Blood and sputum routine cultures at his admission showed no significant growth.

The patient was treated with hydroxychloroquine sulfate, 200 mg tablet (oral use, 400 mg, every 12 h); methylprednisolone acetate, 40 mg/mL injection 1 mL vial (endosinusal use, 40 mg, every 8 h); salbutamol, 100 microgram/actuation inhaler (inhalation use, 2.5 mg, every 8 h); ipratropium bromide anhydrous, 20 microgram/actuation inhaler (inhalation use, 250 mg, every 8 h); azithromycin, 250 mg capsule (oral use, 500 mg, every 24 h); imipenem 500 mg + cilastatin 500 mg injection: powder for, vial + normal saline; cefepime, 2 g injection: powder for, vial + normal saline; paracetamol, 1 g/100 mL injection: intravenous infusion, 100 mL vial; omeprazole, 20 mg capsule (oral use, 40 mg, every 24 h); paracetamol 1 g/100 mL injection: intravenous infusion, 100 mL vial. The patient received care in a non-critical unit for 23 days before being discharged to home.

Patient’s serum taken at admission and preserved at −80 °C was used for retrospective investigation for atypical pneumonia bacterial infection, including *Mycoplasma pneumoniae* and *Chlamydophila pneumoniae*, via Indirect ELISA kits sensitivity 94.3%, specificity 86.5% (G/M1015 and G/M1007, respectively) (Vircell, Granada, Spain) and *Legionella pneumophila* serogroups 1 to 7 using Serion ELISA classic kits ESR106M sensitivity 94%, specificity 96% (Institute Virion\Serion GmbH, Würzburg, Germany). According to the kits manufacturer’s instructions, human IgG sorbent was used to avoid false-positive, false-negative, and possible cross-reactivity results. These tests showed positivity for IgM antibodies against *Mycoplasma pneumoniae* and *Legionella pneumophila*, indicating simultaneous recent acute infections with SARS-CoV-2.

The patient’s data were extracted from his medical records.

## 3. Discussion

In this work, we report on a rare case of a SARS-CoV-2 elderly patient with retrospectively diagnosed double atypical bacterial coinfections (*Legionella pneumophila* and *Mycoplasma pneumoniae*) in the early phase of the pandemic in Saudi Arabia (21 June 2020), when the total cases were 157,612, with 3392 daily new cases and 1267 recorded deaths [2].

Several human organs such as the heart, brain, and kidney have ACE2 receptors on their cells, which is a functional receptor for SARS-CoV-2, making these organs susceptible to SARS-CoV-2 attack [16]. Therefore, CT scan and ECG were applied to investigate possible abnormality in the brain and the heart that might occur due to the virus dissemination and infections. Brain CT scan showed no acute major infraction or hemorrhage (Figure 1), and the ECG displayed no significant changes.

The patient was pre-diagnosed for COVID-19 at the public health service center three days before arriving at the hospital with some common COVID-19 clinical manifestations. However, the patient’s symptoms at admission, such as a productive cough with yellow sputum, a month-long fever, neutrophilia, and bilateral multifocal patchy opacity at the lower zone, indicated possible bacterial coinfections. Nevertheless, routine microbiology cultures from blood and sputum did not help detect bacterial co-pathogens. It is worth mentioning that identifying causative pathogens for pneumonia patients through culturing techniques from blood and respiratory tract samples can reach only 5–10% of the cases, whereas molecular and antigen antibody-based tests can detect the etiological organisms in 50–75% of pneumonia cases [17,18]. Moreover, low lymphocyte counts (lymphopenia) had been observed, which is a characteristic of SARS-CoV2 infection [19].

Several studies had reported that bacterial coinfections among COVID-19 patients had occurred after a few days of hospital admission, and those bacteria were hospital-acquired, multidrug-resistant (MDR) such as *Pseudomonas aeruginosa*, *Klebsiella* spp., *Acinetobacter baumannii*, and methicillin-resistant *Staphylococcus aureus* (MRSA). Most of these patients were critically ill and receiving invasive treatment with catheters, central lines, and auto-ventilation, which might enhance MDR bacteria coinfections [10,14].

The patient’s age (76 years) and diabetes were potential factors for community-acquired bacterial pneumonia [3]. In addition, the prolonged hospital stay for the SARS-CoV-2 patient here might be related to *Legionella pneumophila* and *Mycoplasma pneumoniae* coinfections. It is unclear whether these coinfecting bacteria increased the severity of the SARS-CoV-2 infection, or their infection was worsened by the virus. However, during SARS-CoV-2 infection, neutrophils in the lung produce neutrophil elastase (serine protease), which facilitates COVID-19 cell entry and promotes its infection [20,21].

Nevertheless, the patient was treated according to the Saudi Arabia Ministry of Health guideline for SARS-CoV-2 that included some empirical antibacterial agents, such as azithromycin. Other antibiotics were added (imipenem and cefepime) because of the suspicion of bacterial coinfection despite the negative culture results. However, empirical administration of the antibiotics, as mentioned earlier, seemed to be effective against these organisms, and the effectiveness of empiric atypical bacteria coverage in hospitalized adult patients with CAP has been reported in several studies [22,23]. On the other hand, the patients’ age (≥65 years) and diabetes were also potential risk factors among critically ill SARS-CoV-2, as recent reports in Saudi Arabia [24] have highlighted these conditions, which might have also been attributed to the prolonged hospital stay here.

The overlapping in some clinical manifestations between community-acquired atypical pneumonia and SARS-CoV-2 is a real challenge in distinguishing bacterial coinfection from COVID-19 that might play a role in biased reporting of these sorts of coinfections during the current pandemic.

As a retrospective investigation, some limitations were unavoidable, such as obtaining respiratory samples for confirmatory molecular diagnosis (PCR) for these two atypical bacteria and might help to detect some of their genes responsible for antibiotics resistance, as the over-prescription of macrolides as an empirical treatment during the current pandemic would probably increase their resistance. However, this case report sheds light on the possible incidence of community-acquired bacterial coinfections among elderly COVID-19 patients at higher risk for community-acquired pneumonia.

## 4. Conclusions

This work reported a rare case of SARS-CoV-2 patient in Saudi Arabia coinfected with double community-acquired atypical bacteria, *Legionella pneumophila* and *Mycoplasma pneumoniae*, which had been detected retrospectively. Considering coinfections with atypical bacteria and other community-acquired pathogens among elderly COVID-19 patients at higher risk for community-acquired pneumonia upon hospital admission might support clinicians in choosing appropriate approaches for treatment and management, leading to reduced severe outcomes hospitalization time current pandemic.

## Figures and Tables

**Table 1:** Patient’s lab investigations results.

Variables	Results	Referenced Value
SARS-CoV-2	Positive	
C-reactive protein	Positive	
Neutrophils	11,340/μL	1500–8000/μL
Lymphocytes	370/μL	800–5000/μL
INR	1.07	<1.1
Aspartate aminotransferase (AST), serum	28	0–31 U/L H
Chloride, serum	100	98–106 mmol/L
Glucose, serum	7.10	<6
Creatinine, serum	89.61	53–97.2 µmol/L
Calcium, serum	2.10	2.2–2.7 mmol/L
Direct bilirubin, serum	2.51	3.4–12 µmol/L
Blood urea nitrogen	5.60	2.5–7.1 mmol/L
Prothrombin time, plasma	14.20	11–12.5 s

## Data Availability

Data sharing not applicable.

## References

[1] Lai C.-C., Shih T.-P., Ko W.-C., Tang H.-J., Hsueh P.-R. (2020). Severe Acute Respiratory Syndrome Coronavirus 2 (SARS-CoV-2) and Coronavirus Disease-2019 (COVID-19): The Epidemic and the Challenges. Int. J. Antimicrob. Agents.

[2] Saudi Arabia COVID: 546,163 Cases and 8633 Deaths—Worldometer. https://www.worldometers.info/coronavirus/country/saudi-arabia.

[3] Marrie T.J. (2000). Community-Acquired Pneumonia in the Elderly. Clin. Infect. Dis. Off. Publ. Infect. Dis. Soc. Am..

[4] May D.S., Kelly J.J., Mendlein J.M., Garbe P.L. (1991). Surveillance of Major Causes of Hospitalization among the Elderly, 1988. MMWR. CDC Surveill. Summ. Morb. Mortal. Wkly. Rep. CDC Surveill. Summ..

[5] Lim W.S., Macfarlane J.T., Boswell T.C., Harrison T.G., Rose D., Leinonen M., Saikku P. (2001). Study of Community Acquired Pneumonia Aetiology (SCAPA) in Adults Admitted to Hospital: Implications for Management Guidelines. Thorax.

[6] Zhu X., Ge Y., Wu T., Zhao K., Chen Y., Wu B., Zhu F., Zhu B., Cui L. (2020). Co-Infection with Respiratory Pathogens among COVID-2019 Cases. Virus Res..

[7] Wang M., Wu Q., Xu W., Qiao B., Wang J., Zheng H., Jiang S., Mei J., Wu Z., Deng Y. (2020). Clinical Diagnosis of 8274 Samples with 2019-Novel Coronavirus in Wuhan. medRxiv.

[8] Yang X., Yu Y., Xu J., Shu H., Xia J., Liu H., Wu Y., Zhang L., Yu Z., Fang M. (2020). Clinical Course and Outcomes of Critically Ill Patients with SARS-CoV-2 Pneumonia in Wuhan, China: A Single-Centered, Retrospective, Observational Study. Lancet Respir. Med..

[9] Yue H., Zhang M., Xing L., Wang K., Rao X., Liu H., Tian J., Zhou P., Deng Y., Shang J. (2020). The Epidemiology and Clinical Characteristics of Co-Infection of SARS-CoV-2 and Influenza Viruses in Patients during COVID-19 Outbreak. J. Med. Virol..

[10] Rawson T.M., Moore L.S.P., Zhu N., Ranganathan N., Skolimowska K., Gilchrist M., Satta G., Cooke G., Holmes A. (2020). Bacterial and Fungal Coinfection in Individuals With Coronavirus: A Rapid Review To Support COVID-19 Antimicrobial Prescribing. Clin. Infect. Dis. Off. Publ. Infect. Dis. Soc. Am..

[11] Jang T.-N., Yeh D.Y., Shen S.-H., Huang C.-H., Jiang J.-S., Kao S.-J. (2004). Severe Acute Respiratory Syndrome in Taiwan: Analysis of Epidemiological Characteristics in 29 Cases. J. Infect..

[12] Arabi Y.M., Deeb A.M., Al-Hameed F., Mandourah Y., Almekhlafi G.A., Sindi A.A., Al-Omari A., Shalhoub S., Mady A., Alraddadi B. (2019). Macrolides in Critically Ill Patients with Middle East Respiratory Syndrome. Int. J. Infect. Dis. IJID Off. Publ. Int. Soc. Infect. Dis..

[13] Richardson S., Hirsch J.S., Narasimhan M., Crawford J.M., McGinn T., Davidson K.W., Barnaby D.P., Becker L.B., Chelico J.D. (2020). Northwell COVID-19 Research Consortium. Presenting Characteristics, Comorbidities, and Outcomes Among 5700 Patients Hospitalized With COVID-19 in the New York City Area. JAMA.

[14] Søgaard K.K., Baettig V., Osthoff M., Marsch S., Leuzinger K., Schweitzer M., Meier J., Bassetti S., Bingisser R., Nickel C.H. (2021). Community-Acquired and Hospital-Acquired Respiratory Tract Infection and Bloodstream Infection in Patients Hospitalized with COVID-19 Pneumonia. J. Intensive Care.

[15] Xing Q., Li G., Xing Y., Chen T., Li W., Ni W., Deng K., Gao R., Chen C., Gao Y. (2020). Precautions are Needed for COVID-19 Patients with Coinfection of Common Respiratory Pathogens. medRxiv.

[16] Zhao Y., Zhao Z., Wang Y., Zhou Y., Ma Y., Zuo W. (2020). Single-Cell RNA Expression Profiling of ACE2, the Receptor of SARS-CoV-2. Am. J. Respir. Crit. Care Med..

[17] Lim W.S. (2020). Pneumonia—Overview. Reference Module in Biomedical Sciences.

[18] Jain S., Self W.H., Wunderink R.G., Fakhran S., Balk R., Bramley A.M., Reed C., Grijalva C.G., Anderson E.J., Courtney D.M. (2015). Community-Acquired Pneumonia Requiring Hospitalization among U.S. Adults. N. Engl. J. Med..

[19] Zhao Q., Meng M., Kumar R., Wu Y., Huang J., Deng Y., Weng Z., Yang L. (2020). Lymphopenia Is Associated with Severe Coronavirus Disease 2019 (COVID-19) Infections: A Systemic Review and Meta-Analysis. Int. J. Infect. Dis..

[20] Alfarouk K.O., Al Houfie S.T.S., Ahmed S.B.M., Shabana M., Ahmed A., Alqahtani S.S., Alqahtani A.S., Alqahtani A.M., Ramadan A.M., Ahmed M.E. (2021). Pathogenesis and Management of COVID-19. J. Xenobiotics.

[21] Zuo Y., Yalavarthi S., Shi H., Gockman K., Zuo M., Madison J.A., Blair C.N., Weber A., Barnes B.J., Egeblad M. (2020). Neutrophil Extracellular Traps in COVID-19. JCI Insight.

[22] Eljaaly K., Alshehri S., Aljabri A., Abraham I., Al Mohajer M., Kalil A.C., Nix D.E. (2017). Clinical Failure with and without Empiric Atypical Bacteria Coverage in Hospitalized Adults with Community-Acquired Pneumonia: A Systematic Review and Meta-Analysis. BMC Infect. Dis..

[23] Eliakim-Raz N., Robenshtok E., Shefet D., Gafter-Gvili A., Vidal L., Paul M., Leibovici L. (2012). Empiric Antibiotic Coverage of Atypical Pathogens for Community-Acquired Pneumonia in Hospitalized Adults. Cochrane Database Syst. Rev..

[24] Albalawi O., Alharbi Y., Bakouri M., Alqahtani A., Alanazi T., Almutairi A.Z., Alosaimi B., Mubarak A., Choudhary R.K., Alturaiki W. (2021). Clinical Characteristics and Predictors of Mortality among COVID-19 Patients in Saudi Arabia. J. Infect. Public Health.

